# CT after interhospital transfer in acute ischemic stroke: Imaging findings and impact of prior intravenous contrast administration

**DOI:** 10.3389/fneur.2022.1023147

**Published:** 2022-12-07

**Authors:** Franziska Mueller, Matthias P. Fabritius, Lena Stueckelschweiger, Sophia Kiesl, Sebastian Moench, Steffen Tiedt, Jan Rémi, Lars Kellert, Moriz Herzberg, Clemens Küpper, Konstantinos Dimitriadis, Jens Ricke, Daniel Puhr-Westerheide, Thomas Liebig, Wolfgang G. Kunz, Paul Reidler

**Affiliations:** ^1^Department of Radiology, University Hospital, LMU Munich, Munich, Germany; ^2^Institute for Stroke and Dementia Research, LMU Munich, Munich, Germany; ^3^Department of Neurology, University Hospital, LMU Munich, Munich, Germany; ^4^Department of Diagnostic and Interventional Radiology, University Hospital Wuerzburg, Wuerzburg, Germany; ^5^Department of Neuroradiology, University Hospital, LMU Munich, Munich, Germany

**Keywords:** stroke, CT, perfusion, IV contrast, interhospital transfer

## Abstract

**Objectives:**

Large vessel occlusion (LVO) stroke patients routinely undergo interhospital transfer to endovascular thrombectomy capable centers. Imaging is often repeated with residual intravenous (IV) iodine contrast at post-transfer assessment. We determined imaging findings and the impact of residual contrast on secondary imaging. Anterior circulation LVO stroke patients were selected out of a consecutive cohort. Directly admitted patients were contrast naïve, and transferred patients had previously received IV iodine contrast for stroke assessment at the referring hospital. Two independent readers rated the visibility of residual contrast on non-contrast computed tomography (CT) after transfer and assessed the hyperdense vessel sign. Multivariate linear regression analysis was used to investigate the association of the Alberta Stroke Program Early CT score (ASPECTS) with prior contrast administration, time from symptom onset (TFSO), and CTP ischemic core volume in both directly admitted and transferred patients.

**Results:**

We included 161 patients, with 62 (39%) transferred and 99 (62%) directly admitted patients. Compared between these groups, transferred patients had a longer TFSO-to-imaging at our institution (median: 212 vs. 75 min, *p* < 0.001) and lower ASPECTS (median: 8 vs. 9, *p* < 0.001). Regression analysis presented an independent association of ASPECTS with prior contrast administration (β = −0.25, *p* = 0.004) but not with TFSO (β = −0.03, *p* = 0.65). Intergroup comparison between transferred and directly admitted patients pointed toward a stronger association between ASPECTS and CTP ischemic core volume in transferred patients (β = −0.39 vs. β = −0.58, *p* = 0.06). Detectability of the hyperdense vessel sign was substantially lower after transfer (66 vs. 10%, *p* < 0.001).

**Conclusion:**

Imaging alterations due to residual IV contrast are frequent in clinical practice and render the hyperdense vessel sign largely indetectable. Larger studies are needed to clarify the influence on the association between ASPECTS and ischemic core.

## Key messages

### What is already known on this topic

Currently, the influence of prior intravenous iodinated contrast administration on imaging appearance and Alberta Stroke Program Early CT score (ASPECTS) scoring in the setting of transferred stroke patients is unknown.

### What this study adds

We present a comprehensive evaluation of secondary imaging of transferred stroke patients at a comprehensive stroke center. Common imaging findings include residual intravascular contrast and obscuration of the hyperdense vessel sign. Correlation of ASPECTS and computed tomography (CT) perfusion-based ischemic core might differ compared to patients without prior contrast administration.

### How this study might affect research, practice, or policy

This study aims at raising awareness among neuroradiologists on how residual contrast in transferred stroke patients can lead to imaging alterations and to ensure correct interpretation.

## Background

Endovascular thrombectomy (EVT) has become the standard of care in large vessel occlusion (LVO) stroke. However, only ~10% of certified stroke centers in the US are comprehensive stroke centers (CSCs) with EVT capabilities (1). After initial clinical and imaging assessment at a primary stroke center (PSC), patients are often transferred to CSCs for further treatment evaluation and monitoring. Approximately 25% of acute ischemic stroke patients at CSCs are transferred (2). In extended time windows, this number is even higher, with 59 and 66% transferred patients in the DAWN and DEFUSE 3 study (3–6).

Imaging assessment at PSCs typically includes non-contrast computed tomography (CT), and an increasing number of publications also recommend CT angiography (CTA) with intravenous (IV) iodine contrast (7, 8). Secondary imaging at CSCs often repeats the performed examinations and complements CT perfusion (CTP) analysis (8, 9). This is even the case in direct-to-angiosuite approaches with pre-therapeutic imaging using flat-panel CT (10, 11).

At both imaging sites, the visual 11-point (from 0 to 10) Alberta Stroke Program Early CT Score (ASPECTS) on non-contrast CT is usually determined, which includes the regional scoring of early ischemic changes such as loss of gray-white matter distinction and focal swelling in the middle cerebral artery (MCA) territory (12). An ASPECTS of ≥6 is the current guideline-based cutoff for EVT (13). ASPECTS decay during inter-hospital transfer is frequent and led to EVT ineligibility of approximately one-third of transferred patients in the study by Mokin et al. (14, 15).

Since more and more patients undergo imaging assessment including the injection of IV iodine contrast for CTA at PSCs as mentioned above, an increasing number of transferred patients is not contrast naïve on secondary imaging at CSCs due to recirculating IV contrast (16). Iodine contrast agents used for CT imaging are eliminated by the kidneys with a half-life of ~90–120 min (17). In patients with decreased renal function, elimination can be further delayed. Therefore, residual contrast is usually present when imaging after the transfer is conducted and might therefore influence imaging characteristics and evaluation. For CTP, a recent study found biasing influence of recent IV contrast administration on automated ischemic core estimation in a small sample size (18). The impact of recirculating IV contrast on secondary non-contrast CT and ASPECTS evaluation has not yet been examined to the best of our knowledge.

Therefore, we aimed to describe the findings of residual IV contrast on secondary CT after inter-hospital transfer and further examined the impact on the relation between ASPECTS and CTP ischemic core.

## Methods

### Study design and population

This retrospective study was approved by the institutional review board of LMU Munich according to the Declaration of Helsinki of 2013, and the requirement for written informed consent was waived. Patients with acute ischemic stroke due to LVO of the anterior circulation were selected out of a consecutive cohort of prospectively enrolled patients. All patients were treated with EVT between 2015 and 2020. Patients were either directly admitted or transferred from external PSCs to our institution. In total, 23 PSCs transferred patients to our hospital.

For our retrospective analysis, we included patients with:

(1) internal carotid artery, M1 or M2 segment artery occlusion.
(2) complete dataset with non-contrast CT, CT angiography, and CTP imaging on admission at our institution.
(3) known time from symptom onset (TFSO).

We excluded patients:

(1) without prior IV iodine contrast administration for stroke assessment (CTA and/or CTP) at PSC in case of interhospital transfer.
(2) with prior IV iodine contrast administration within the last 24 h in case of an in-house stroke.

### Clinical data and interhospital transfer

Glomerular filtration rate (GFR) was based on blood samples drawn on admission at our institution using the CKD-EPI formula (19). Interhospital transfer status including imaging modality at PSCs, external IV thrombolysis status, and transfer times were taken from medical records. Imaging data from PSCs were only available in very few cases and could not be systematically evaluated.

### Multiparametric CT imaging and analysis

The imaging protocol at our institution included non-contrast CT, CTA, and CTP. Examinations were performed on CT scanners of the same vendor (SOMATOM Force, SOMATOM Definition AS+, SOMATOM Definition Flash, Siemens Healthineers, Forchheim, and Germany). Non-contrast CT parameters included 100 kV tube voltage and applied tube current modulation (X-Care) and 0.6 mm collimation. CTP data were processed using Syngo Neuro Perfusion CT (Siemens Healthineers, Forchheim, Germany) including automated calculation of ischemic core and penumbra volumes according to the manufacturer's thresholds (CBV <1.2/100 ml and CBF <35.1 ml/100 ml/min) (20). We defined total ischemic volume as the sum of penumbra and core volumes.

Non-contrast CT ASPECTS, clot burden score, regional leptomeningeal (rLM) collateral score, and presence of hyperdense artery sign were determined by two independent readers (W.G.K. and P.R.) at admission imaging blinded to clinical data and transfer status. ASPECTS reading was performed with standardized window settings of center/width: 40/40 HU and using reconstructions with 5 mm slice thickness. Two different independent readers (M.P.F., D.P.-W.) evaluated subjective parameters of visibility of residual contrast on non-contrast CT after interhospital transfer on a four-point Likert scale (increments: none, low, medium, and high). For this reading, we used the parameters' overall image alteration by residual contrast, alteration of parenchymal appearance by residual contrast defined as altered gray-white matter differentiation, and visibility of intravascular contrast in the intracranial arterial and venous system defined by the impression of vascular hyperdensity. In case of disagreement, the consensus was reached in a separate session.

### Statistical analysis

Analyses were performed using SPSS Statistics 23 (IBM, Armonk NY 2016, commercial software) and R version 3.6.2 (R Foundation for Statistical Computing, Vienna Austria). Proportion analysis tests for categorical variables were performed using the *x*^2^ test. Non-parametric tests were performed using the Mann-Whitney *U* test, and for ordinal variables using the independent samples median test.

Linear regression analysis was performed for non-contrast CT ASPECTS and CTP ischemic core volume adjusted for TFSO in the direct admission and interhospital transfer group. The difference of resulting regression coefficients was tested for significance using the interaction term transfer status × ischemic core volume as applied in other studies (21). Multivariable linear regression analysis was performed in all patients to further identify associations of non-contrast CT ASPECTS on admission with clinical and imaging parameters using transfer status as an independent variable. All regression analyses were performed after the exclusion of 4 outliers (1 in the transfer group and 3 in the direct admission group) with large core but high ASPECTS. To account for potential heteroskedasticity in our data, we used robust (Huber-White) standard errors in all models. To avoid overfitting of the regression models, we tested the multicollinearity of independent variables using the variance inflation factor (VIF). Inter-reader reliability was determined by calculation of the intraclass correlation coefficient (ICC) and Cohen's kappa. A statistical significance was defined as *p* < 0.05.

## Results

### Patient characteristics

Notably, 161 patients were included, out of which 99 (62%) were directly admitted and 62 (39%) were transferred patients. As expected, transferred patients had a significantly longer TFSO to imaging at our institution (median [interquartile range (IQR)]: 212 [168–261] vs. 75 min [60–106], *p* < 0.001) and lower ASPECTS at admission imaging (median [IQR]: 8 [6–9] vs. 9 [8–10], *p* < 0.001). The observed larger median ischemic core size of transferred patients did not reach statistical significance (median [IQR]: 40 [25–82] vs. 34 ml [21–56), *p* = 0.07). Detailed patient characteristics are presented in Table 1.

**Table 1:** Patient characteristics.

	**Overall**	**Direct admission**	**IT with prior IV contrast**	***p*-value**
	**(*N* = 161)**	**(*n* = 99)**	**(*n* = 62)**	
**Patient data**				
Age	74 (63–81)	75 (64–83)	74 (63–79)	0.24
Male sex	92 (57%)	56 (57%)	36 (58%)	0.85
Female sex	69 (43%)	43 (43%)	26 (42%)	0.85
Time from symptom onset [min]	122 (70–209)	75 (60–106)	212 (168–261)	**< 0.001**
Transfer time [min]			120 (90–143)	**–**
NIHSS on admission	14 (8–18)	12 (8–17)	16 (9–20)	0.09
GFR [ml/min]	79 (56–90)	79 (55–90)	76 (57–91)	0.79
**Treatment**				
IV thrombolysis	117 (73%)	73 (74%)	44 (71%)	0.70
EVT	161 (100%)	99 (100%)	62 (100%)	–
**Imaging**				
ASPECTS	9 (7–10)	9 (8–10)	8 (6–9)	**< 0.001**
Occluded vessels*				
ICA	36 (22%)	24 (24%)	12 (19%)	0.47
Carotid T	35 (22%)	24 (24%)	11 (18%)	0.33
M1 segment of MCA	121 (75%)	76 (77%)	45 (73%)	0.55
M2 segment of MCA	52 (32%)	33 (33%)	19 (31%)	0.92
Ischemic core volume [mL]	35 (21–60)	34 (21–56)	40 (25–82)	0.07
Total ischemic volume [mL]	187 (153–231)	186 (153–230)	188 (145–234)	0.90
rLM collateral score	16 (12–18)	16 (12–18)	16 (12–18)	0.87
Clot burden score	6 (4–8)	6 (4–8)	7 (4–8)	0.80
Hyperdense vessel sign visible	71 (44%)	65 (66%)	6 (10%)	**< 0.001**
Hemorrhage on CSC admission	5 (3%)	0 (0%)	5 (8%)	**0.01**

Values presented are count (percentage) for categorical and median (interquartile range) for ordinal or continuous variables. Proportion analysis tests for categorical variables were performed using the *x*^2^ test. Non-parametric tests for non-normally distributed continuous variables were performed using the Mann-Whitney U test and for ordinal variables using the independent samples median test. Bold numbers indicate p-values < 0.05.

* Occlusions were rated in all affected parts of the anterior circulation; therefore, numbers and percentages may exceed 100%.

IT, interhospital transfer; IV, intravenous; NIHSS, national institutes of health stroke scale; ASPECTS, alberta stroke program early CT score; ICA, internal carotid artery; ICH, intracranial hemorrhage; GFR, glomerular filtration rate; EVT, endovascular thrombectomy; MCA, middle cerebral artery; rLM, regional leptomeningeal; CSC, comprehensive stroke center.

### Imaging analysis and findings after interhospital transfer

Overall altered image impression due to residual IV contrast was rated high in 39% and medium in 47% of transferred cases. This largely overlaps with the rating of intravascular contrast visibility, which was medium in 39% and high in 37% of cases. Alteration of parenchymal image impression by residual contrast was less frequent with medium and high visibility in only 3% of cases each.

Although no significant difference in the rate of occlusion location was present, a hyperdense vessel sign was visible in only 6 (10%) of transferred patients but in 65 (66%) of the directly admitted patients (*p* < 0.001). Hemorrhage was present in 5 (8%) transferred patients and in none of the directly admitted patients (*p* = 0.01). There were no space-occupying hemorrhages.

**Figure 1:**
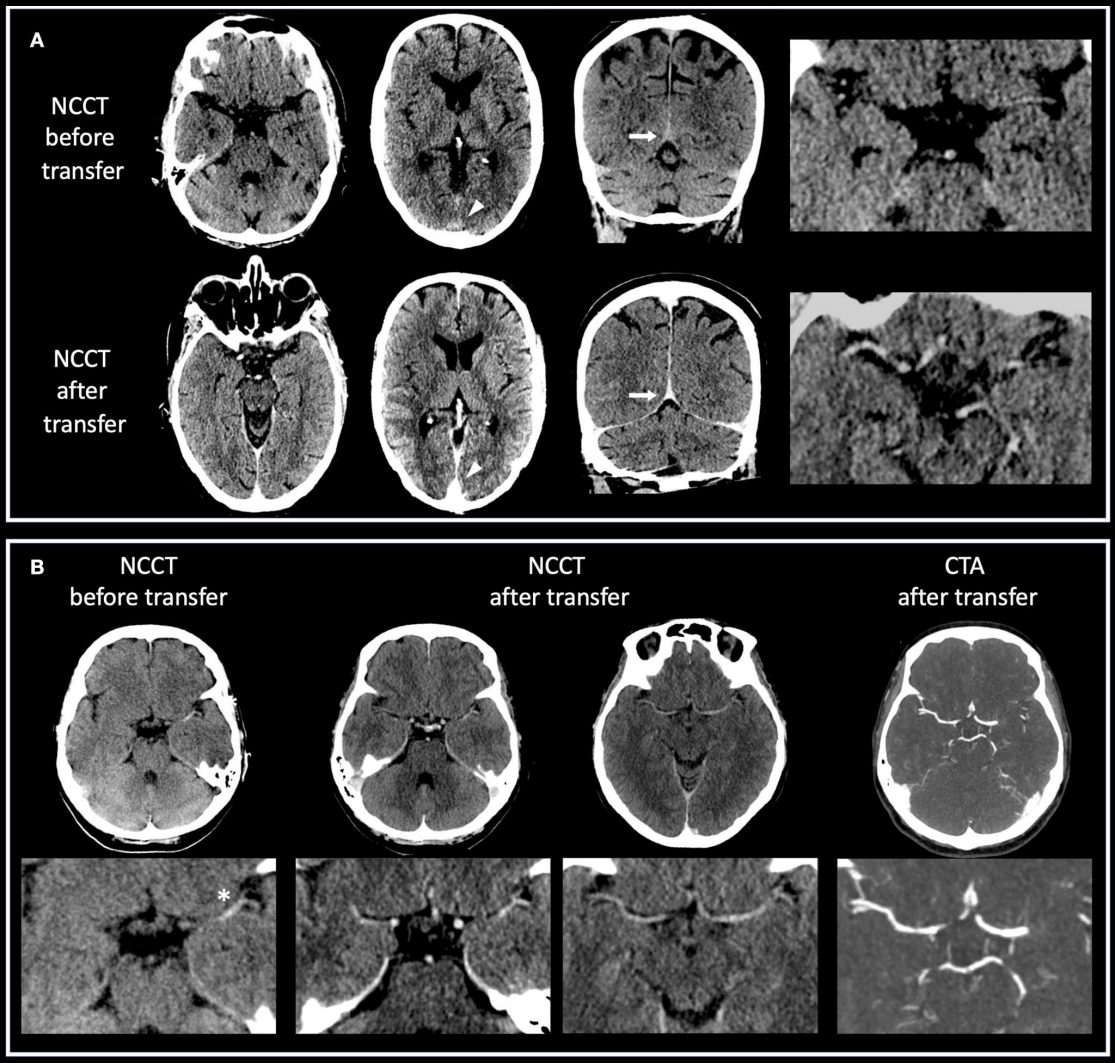
**(A,B)** Case examples of non-contrast CT imaging before and after interhospital transfer. **(A)** Patient with right-sided ICA occlusion who underwent CT imaging at PSC including CTA. Transfer time was 85 min, and kidney function was normal. Residual IV iodine contrast produces hyperdense intracranial vessels after the transfer, especially around the Circle of Willis (axial and enlarged views) and in sinuses on axial (arrowheads) and coronal (arrows) views. **(B)** Patient with left-sided M1 occlusion who underwent CT imaging at PSC including CTA. Transfer time was 90 min, and kidney function was normal. Presented are axial and enlarged views. Before transfer, a hyperdense vessel sign (asterisk) due to thrombotic occlusion is clearly visible on non-contrast CT. After transfer, intracranial vessels are hyperdense due to residual IV iodine contrast, which obscures intravascular thrombus. CTA after transfer confirms persistent vessel occlusion.

Detailed results are displayed in Tables 1, 2. Imaging examples are provided in Figure 1.

**Table 2:** Imaging alterations due to residual IV iodine contrast on non-contrast CT after CSC arrival in the transfer group (*n* = 62).

	**None**	**Low**	**Medium**	**High**
Overall image alteration	1 (2%)	8 (13%)	29 (47%)	24 (39%)
Alteration of parenchymal appearance	51 (82%)	7 (11%)	2 (3%)	2 (3%)
Visibility of residual intravascular contrast	2 (3%)	13 (21%)	24 (39%)	23 (37%)

Two independent readers evaluated non-contrast CTs after CSC arrival in patients with interhospital transfer and prior IV iodine contrast for stroke assessment at the PSC. Changes were rated on a 4-point Likert scale (increments: none, low, medium, and high). Alteration of parenchymal appearance was defined as altered gray-white matter differentiation. IV, intravenous; CSC, comprehensive stroke center; primary stroke center.

Inter-reader reliability was medium to good for ASPECTS (ICC [IQR]: 0.75 [0.66–0.82]), excellent for overall image alteration and intravascular contrast visibility (ICC [IQR]: 0.89 [0.82–0.94] and 0.91 [0.85–0.95]), and medium for parenchymal alteration (ICC [IQR]: 0.69 [0.48–0.81]) (22). Inter-reader agreement for hyperdense vessel sign was substantial (Cohen's kappa: 0.78, 95%-CI: 0.68–0.87).

### Association between non-contrast CT ASPECTS and CTP ischemic core

Multivariate linear regression included non-contrast CT ASPECTS as dependent and age, sex, TFSO, GFR, transfer status, CTP ischemic core volume, rLM collateral score, and clot burden score as independent variables. Furthermore, IVT status before transfer and transfer time was included in a separate analysis of the transfer group. Transfer status (β = −0.25, *p* = 0.004), CTP ischemic core volume (β = −0.41, *p* < 0.001), and clot burden score (β = 0.22, *p* = 0.002) presented the independent association with ASPECTS. TFSO (β = −0.04, *p* = 0.64) and other parameters including visual overall image alteration did not display significant influence (*p* > 0.05). When comparing groups, we performed separate linear regression analyses with ASPECTS as dependent and CTP ischemic core volume and TFSO as independent variables. In both groups, CTP ischemic core volume presented independent association (direct admission: β = −0.51, *p* = 0.002; transfer group: β = −0.62, *p* < 0.001) without significant influence of TFSO (*p* > 0.05). The difference between both regression coefficients using an interaction term was borderline non-significant using robust standard errors (*p* = 0.058) but significant using standard analysis (*p* < 0.001). VIF was found below the critical value of 3.3 for all analyses (23). Results of the regression analysis are displayed in Table 3.

**Table 3:** Multivariate regression analysis for association of non-contrast CT ASPECTS at CSC arrival with clinical and imaging parameters.

**Independent variables (*n* = 157)**	**β**	***p*-value**	**VIF**
Age	−0.06	0.35	1.59
Sex	−0.02	0.69	1.15
Time from symptom onset	−0.04	0.64	1.47
GFR	−0.01	0.88	1.53
Admission NIHSS	−0.05	0.46	1.28
Interhospital transfer with prior intravenous contrast	−0.25	**0.004**	1.53
CTP ischemic core volume	−0.41	**< 0.001**	1.64
rLM collateral score	0.09	0.28	1.92
Clot burden score	0.22	**0.002**	1.48
IVT before transfer (*n* = 62)^a^	−0.16	0.09	1.45
Transfer time (*n* = 62)^a^	−0.19	0.06	1.71
Overall image alteration (*n* = 62)^a^	0.31	0.13	1.02

A multivariate linear regression analysis was performed for the indicated parameters. p-values < 0.05 indicate statistical significance. (a) Only tested in the transfer group (n = 62) with additional independent variables time from symptom onset, GFR, CTP ischemic core volume, rLM collateral score, and clot burden score.

CSC, comprehensive stroke center; VIF, variance inflation factor; GFR, glomerular filtration rate; NIHSS, national institutes of health stroke scale; CTP, CT perfusion; rLM, regional leptomeningeal; IVT, intravenous thrombolysis.

## Discussion

To the best of our knowledge, this is the first study to investigate the impact of recirculating IV contrast on non-contrast CT for acute anterior circulation LVO stroke. We determined decisive alterations of imaging appearance due to residual contrast in most cases after interhospital transfer. Readers reported a medium to high alteration of imaging appearance in the majority of cases after transfer with prior IV contrast administration. This was largely driven by the visibility of residual intravascular contrast. We also attributed the significantly lower detection rate of hyperdense vessel signs in the transfer group to obscuration by residual intravascular contrast. ASPECTS on non-contrast CT at our institution presented a significant association with transfer status but not with TFSO, indicating an influence of prior IV contrast administration. This is further supported by the significant difference in association strength between ASPECTS and ischemic core, which was stronger in the transfer group. Although IV contrast underlies renal elimination, we could not find a significant modification of our findings by renal function or transfer time.

“Non-contrast CT” represents the most widely used imaging acquisition in stroke management. In the light of our work, “non-contrast” foremost describes the technical acquisition. As we have shown, residual contrast after prior IV administration alters image appearance; therefore, contrast-naïve and not contrast-naïve CT would be more appropriate terms.

The rate of patients with recirculating IV contrast on arrival at CSCs will likely increase in the near future as CTA has been declared an imaging standard at PSCs for suspected LVO stroke (7, 8, 16, 24). This causes uncertainty for the use of ASPECTS, which was initially validated for the contrast-naïve situation (12). Assuming a strengthened association between ischemic core and non-contrast naïve CT ASPECTS, this would indicate a potential bias for EVT decisions. A larger ASPECTS decrease in reference to ischemic core volume might preclude patients from EVT when CTP imaging is not performed. Interestingly, approximately one-third of patients in a study by Mokin et al. became EVT ineligible due to ASPECTS decay after interhospital transfer but some were still treated with EVT due to favorable perfusion profiles (14). In the extended time window, as stated by the sub-analysis of the DAWN study, the rate of patients who became ineligible during transfer was not examined, leaving a critical knowledge gap for EVT decision (5).

Potential mechanisms of bias include changes in the visibility of cerebral edema formation (25) or impact on the estimation of ischemic core volumes. While analysis indicates that visible alterations do not directly affect ASPECTS reading, however, this does not exclude edema obscuration after IV contrast. A recent study reported bias of prior IV contrast administration on the CTP core volume estimation by RAPID (RAPID, iSchemaview) in 23 cases, with frequent core estimation around 0 ml (18). ASPECTS was used as a reference standard, but the bias of residual contrast on non-contrast CT was not considered. We could not reproduce this interesting finding in our study; however, we also used different software, which largely estimated coherent core volumes in both groups.

Regarding temporal influence on ASPECTS, Nannoni et al. (26) recently reported a strengthened association between ASPECTS and ischemic core in patients in the extended time window >6 h compared with the overall population, however, not considering the impact of prior IV contrast administration. As our study only included 7 patients presenting later than 6 h after symptom, a significant impact on our results by the implicated effects seems less likely. We further tried to rule out the potential bias of TFSO on our results by including only patients with known TFSO and incorporating this parameter into our regression models. In this study, TFSO did not display a significant influence on ASPECTS despite the significant differences in time to imaging between direct admission and transferred groups.

As a practical clinical point, it needs to be clearly stated that a hyperdense vessel sign on not contrast naïve CT should not be expected in LVO stroke patients as hyperdense thrombi are masked by residual intravascular contrast. In case of insecurity of persistent LVO after the transfer, repeat CTA should therefore not be omitted. Furthermore, the rate of hemorrhages was significantly higher after transfer. A clear distinction between extravasation and real hemorrhage was not possible in our study protocol; therefore, this finding needs further dedicated analysis.

Our results need to be interpreted in light of the study's limitations. First, we provided a limited retrospective sample. We could not gather a more appropriate control group of transferred patients without prior IV contrast administration, as almost all patients who were transferred to our institution received CTA imaging at PSCs. Therefore, we considered TFSO in all our statistical models, which in return shifted our study sample to an earlier time window. Therefore, our results need dedicated replication in the extended time window or patients with unknown onset, also incorporating more patients with low ASPECTS (< 6).

Second, image acquisition and post-processing relied on a single vendor (Siemens Healthineers). Therefore, translatability to other vendors and centers requires further validation. Additionally, the use of virtual non-contrast reconstruction of dual-energy CT data was not explored in our study (27).

Third, visual ASPECTS rating as performed in our study presents different degrees of inter-reader reliability in the literature (28, 29). Also, the determination of residual contrast relied on subjective image impression and did not use a standardized measure. Although a software-based or quantitative assessment might provide more reproducible results in future studies, our approach reflects current clinical practice in the decision process for EVT. Furthermore, we are not able to provide a true gold standard for the presence of a hyperdense sign after the transfer, and therefore used the rate of CTA occlusion as a comparison to deduce the impact of prior contrast.

Finally, in the digital age with increasing data-sharing capabilities and the ability to rapidly copy and send digital imaging from the PSCs to CSCs, the need for repeated imaging might decrease in the future. However, this is highly dependent on geographical area, organization of the healthcare system, and the availability of secure data-sharing platforms. Secondary imaging for EVT evaluation at the CSC is still common, and it is therefore still important for neuroradiologists to be aware of how residual contrast in transferred stroke patients can lead to imaging alterations.

## Conclusion

Residual IV iodine contrast after interhospital transfer alters the visual appearance of non-contrast CT at the CSC in most cases. At present, we cannot deduce a relevant impact on therapy decisions through greater ASPECTS decrease in reference to ischemic core volume. Further analysis in larger data samples is warranted, and potential bias should raise suspicion in case of borderline ASPECTS.

## Data availability statement

The raw data supporting the conclusions of this article will be made available by the authors, without undue reservation.

## Ethics statement

The studies involving human participants were reviewed and approved by Institutional Review Board of the LMU Munich. Written informed consent for participation was not required for this study in accordance with the national legislation and the institutional requirements.

## Author contributions

FM and MF: conceptualization, methodology, formal analysis, investigation, and writing—original draft. LS: formal analysis, investigation, and writing—original draft. SK and DP-W: formal analysis, investigation, and writing—original draft. SM, ST, and JRé: writing—review and editing. LK, KD, JRé, MH, CK, JRi, TL, and WK: conceptualization and writing—review and editing. PR: conceptualization, statistical analysis, and supervision. All authors contributed to the article and approved the submitted version.
